# Supplementary figures and images for: Effects of Maternal Low-Protein Diet on Microbiota Structure and Function in the Jejunum of Huzhu Bamei Suckling Piglets
Source: Animals (Basel). 2019 Sep 23;9(10):713. doi: 10.3390/ani9100713 (PMC6826398; doi:10.3390/ani9100713)

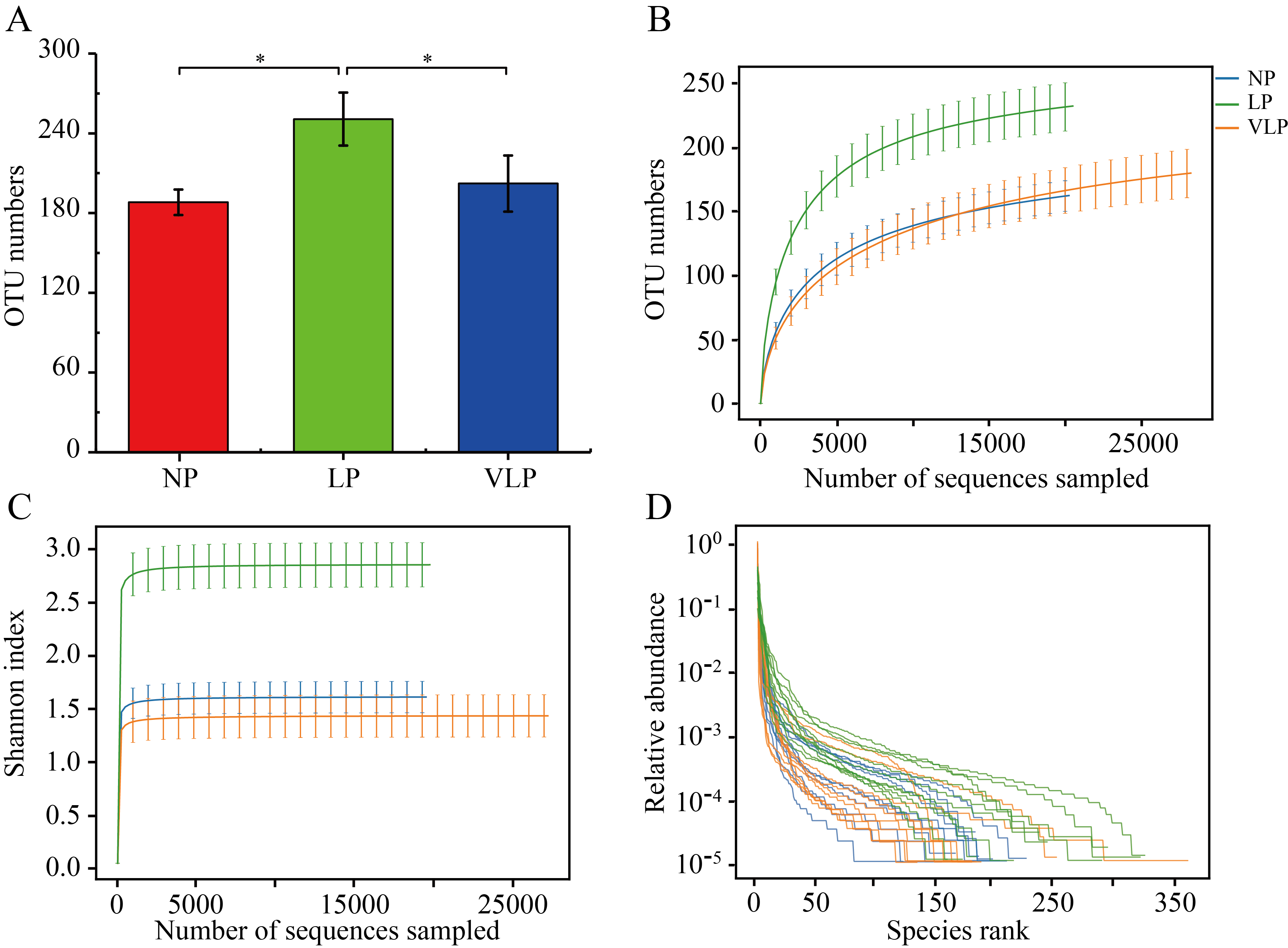

Supplement: Supplementary file 1 [file animals-09-00713-s001.zip › animals-549084-SI/animals-549084-SI/Supplemental Figure S1.png]

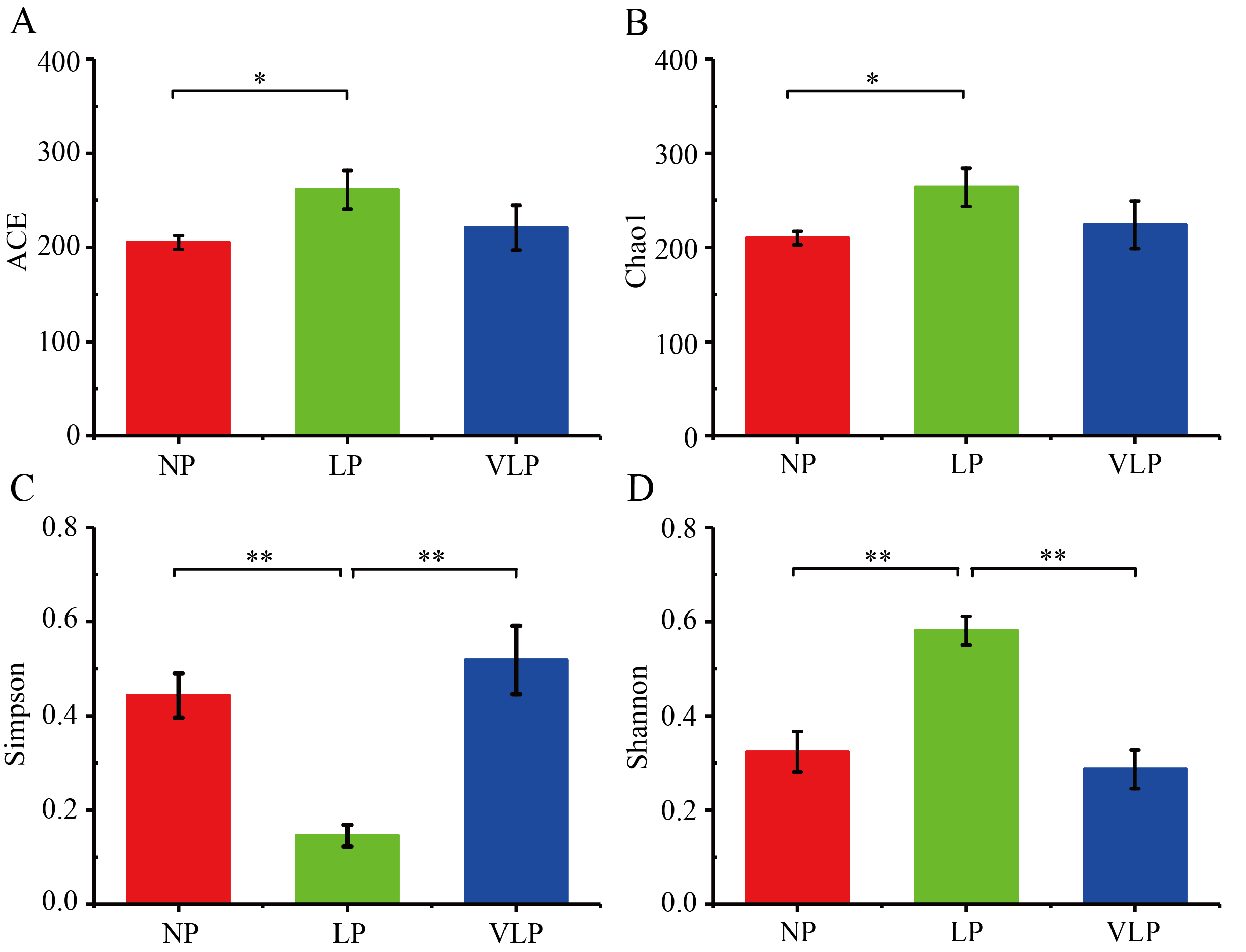

Supplement: Supplementary file 1 [file animals-09-00713-s001.zip › animals-549084-SI/animals-549084-SI/Supplemental Figure S2.png]

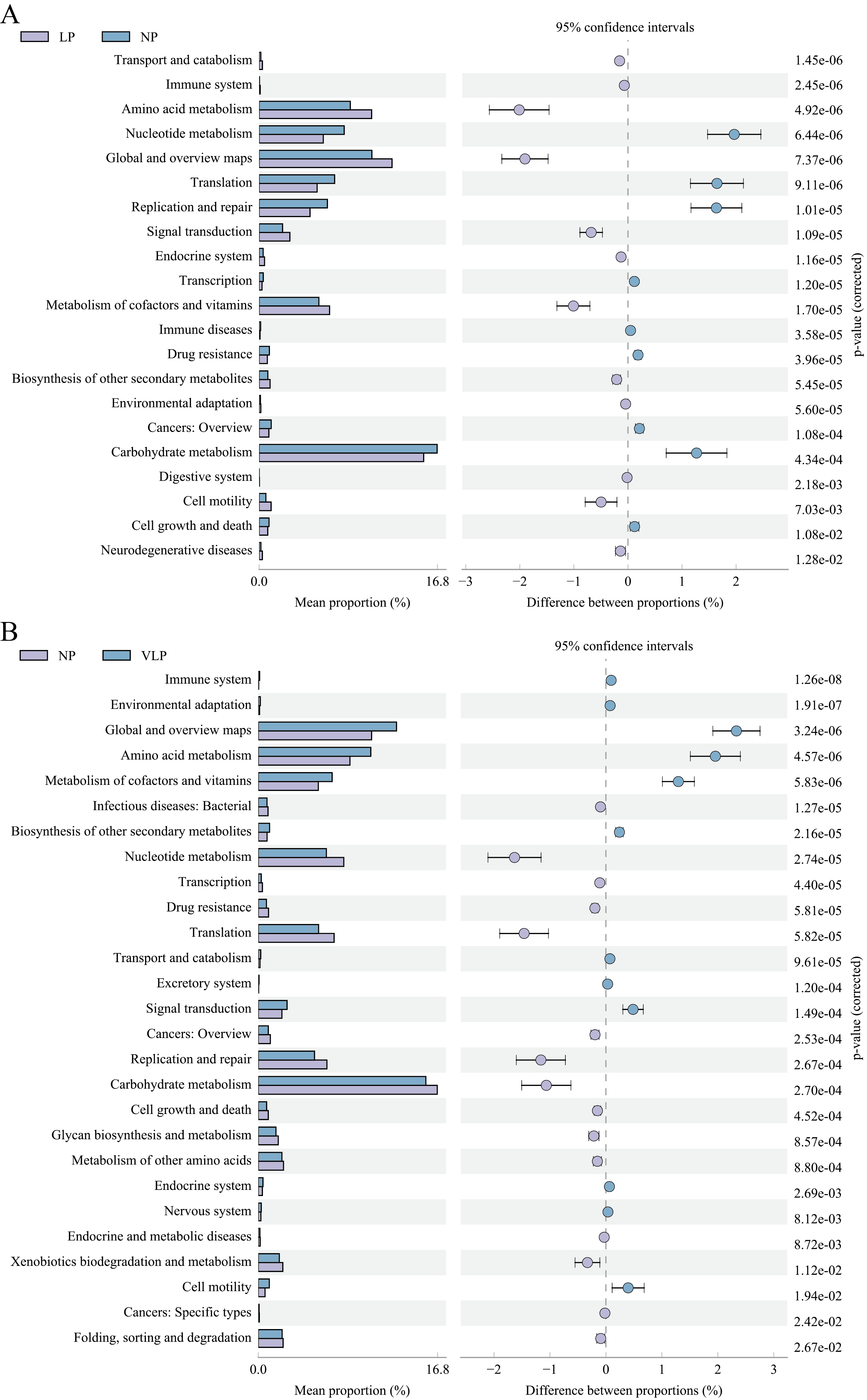

Supplement: Supplementary file 1 [file animals-09-00713-s001.zip › animals-549084-SI/animals-549084-SI/Supplemental Figure S3.png]
